# Counterintuitive consequences of COVID-19 on healthcare workers: A meta-analysis of the relationship between work engagement and job satisfaction

**DOI:** 10.3389/fpsyg.2022.962830

**Published:** 2022-10-10

**Authors:** Bora Yildiz, Tayfun Yildiz, Mustafa Ozbilgin, Harun Yildiz

**Affiliations:** ^1^Department of Management, Faculty of Economics, Istanbul University, Istanbul, Turkey; ^2^Brunel Business School, Organisations and People, College of Business, Arts and Social Sciences, Brunel University London, Uxbridge, United Kingdom; ^3^Department of Organizational Psychology, School of Business, Economics and Informatics, University of London-Birkbeck College, London, United Kingdom; ^4^Department of Management and Organization, Faculty of Economics and Administrative Sciences, Ardahan University, Ardahan, Turkey; ^5^Department of International Trade, Omer Seyfettin Faculty of Applied Sciences, Bandirma Onyedi Eylul University, Balikesir, Turkey

**Keywords:** healthcare, job satisfaction, meta-analysis, work engagement, COVID-19

## Abstract

**Background:**

Studies conducted in the health sector have determined a positive relationship between job satisfaction and work engagement. However, this paper reveals that this relationship turns into a negative or non-significant relationship during the COVID-19 pandemic. We explore the reasons for inconsistency in research findings in this critical period through a meta-analysis.

**Methods:**

This study was conducted according to the PRISMA guidelines and PICO framework. Online databases including Web of Science, Scopus, PubMed, ProQuest, Google Scholar, and additional records from other databases were searched without any time limitation, and all studies published in English that reported the correlation between work engagement and job satisfaction were included in the analysis. In total, 36 individual correlation coefficients were synthesized. R statistical language was used to analyze the data.

**Result:**

A total of 36 studies with a sample size of 16,087 were synthesized. The overall effect size was found as *r* = 0.57 (95% CI [0.50–0.64]). While the moderating effect of national culture was not statistically significant, presence of COVID-19 as the significant moderator explained 37.08% of effect size heterogeneity. Such that the presence of COVID-19 has transformed the positive relationship between work engagement and job satisfaction into a negative but statistically non-significant relationship.

**Conclusion:**

This study empirically challenges the existing assumptions about the positive link between work engagement and job satisfaction. The results of the research can be a guide for managers and policymakers. Specifically, based on these results, different mechanisms can be put in place to support work engagement and, in turn, job satisfaction in the COVID-19 process.

## Introduction

The ongoing COVID-19 pandemic has caused radical changes in the ordinary routines of life. Undoubtedly, health sector workers are the worst hit in the COVID-19 pandemic (Ehrlich et al., 2020; Pappa et al., 2020; Yildiz and Elibol, 2021). Excessive, intense, restless work programs and compulsory labor practices of the governments have put immense pressure on healthcare workers' perceptions, attitudes, and behaviors toward their professions (Duarte et al., 2020; Ardebili et al., 2021; Özmen et al., 2021). The imbalance between home life and work life throughout this process emerged and employees began to burn out with the prolonged pandemic conditions (Lulli et al., 2021). Interactions with colleagues deteriorated due to social distance rules, and the workload and distractions created by the home environment negatively affected the focus of employees on work (Moretti et al., 2020). The risk of infecting themselves and their families because of workers' work on the front lines also negatively impacted their mental health and psychological well-being (Giorgi et al., 2020; Zhang et al., 2021). Physical and emotional reactions to occupational stress from the pandemic ranged from low mood to suicidal thoughts (Stansfeld and Candy, 2006; Lulli et al., 2021). Work engagement (WE) and job satisfaction (JS) are among the variables affected by this challenging context. For example, a positive link between WE and JS of healthcare professionals (Lin et al., 2020; Ng et al., 2021; Sarfaraz et al., 2022) turned negative or nonsignificant in the ongoing COVID-19 process (Gimenez-Espert et al., 2020; Stover, 2020). World Health Organization (2022) calls for nation-states to take adequate measures to study and struggle the negative consequences of the COVID-19 pandemic on healthcare workers, which are the most adversely affected community of professionals in the pandemic. The chain effect of these negative changes in the work engagement of healthcare professionals on patient safety is another salient dimension. Thus, the changes in WE and JS impacted employees as well as quality of care in a way that threatens patient safety (Scott et al., 2022).

Job Demands-Resource (JD-R) model (Nguyen et al., 2018; Gong et al., 2020) is widely accepted theoretical lens to determine the relationship between JS and WE. The model emphasizes that job resources are the basis for WE and why engagement is vital for positive outcomes (Demerouti et al., 2001; Hakanen and Roodt, 2010; Schaufeli and Taris, 2014; Schaufeli, 2017). Past studies indicated that, unlike other job attitudes, WE plays a strategic role in job performance (Christian and Slaughter, 2007; Christian et al., 2011) and sustainable competitive advantage (Eldor, 2016). Grounded on these views and the JD-R model, by creating a holistic view with this meta-analytic study, we tried to support policymakers and researchers in formulating operational strategies and practices that aimed to manage WE and JS effectively. We also tried to determine how COVID-19, as a situational factor, affects the relationship between WE and JS.

Numerous meta-analyses on WE and JS in the healthcare sector have been published. This meta-analysis differs from previous meta-analyses in several ways. First, in the earlier meta-analyses, healthcare workers were not generally evaluated; instead, they focused on the specific healthcare professions such as nursing (Lu et al., 2019; Yildiz and Yildiz, 2022). Secondly, studies on JS or WE were conducted to examine the antecedents and consequences of these variables separately instead of studying the relationship between WE (Halbesleben, 2010) and JS (Lu et al., 2019). Lastly, since the previous meta-analyses were conducted before the COVID-19 (Halbesleben, 2010; Lu et al., 2019), the publications with opposite results that caused the emergence of this study were published during the pandemic period. Therefore, this study's main point from previous meta-analyses is that it brings together studies on healthcare workers both during and before the pandemic.

This study analyzes the existing knowledge about study variables through meta-analyses by testing the moderating effect of the presence of COVID-19. Accordingly, this study aims to (a) specify the overall effect size of the correlation between study variables and (b) analyze the moderating effect of the presence of COVID-19 on the overall effect size. The study reveals counterintuitive findings that challenged the previously established relationship between WE and JS, showing that the COVID-19 pandemic had an adverse impact changing the relationship between WE and JS to negative. We explain why this is and suggest future research directions in the conclusion section.

### Job demands-resources theory and conceptual relationships

Work engagement (WE) is one of the most critical drivers of positive organizational behavior outcomes. Leiter and Bakker (2010) defined WE as “a positive, fulfilling, affective-motivational state of work-related well-being that can be seen as the antipode of job burnout.” As Bakker et al. (2014) emphasized, WE is of vital importance in today's organizations in terms of improving performance, creativity, extra-role behaviors, job and customer satisfaction. In line with these explanations, it can be said that WE is one of the indispensable factors for organizations to achieve their strategic or daily goals together with their employees.

One of the positive and vital consequences of WE is JS (Sonnentag et al., 2012; Moura et al., 2014; Gong et al., 2020). Job satisfaction is defined as “how people feel about their jobs and different aspects of their jobs. It is the extent to which people like or dislike their jobs” (Spector, 1997; p. 2). A meta-analytic study's results indicated that JS was significantly related to psychological (burnout, self-esteem, anxiety, depression) and physical health (Faragher et al., 2005). While JS diminishes mental and physical health problems, dissatisfaction leads to such issues.

The research models on WE are mainly attributed the Job Demands-Resource (JD-R) model (Demerouti et al., 2001; Hakanen and Roodt, 2010; Schaufeli and Taris, 2014; Schaufeli, 2017). Considering the JD-R model, job demands describe “characteristics of the job that potentially evoke strain, if they exceed the employee's adaptive capability” (Bakker et al., 2007; p. 275). Some factors such as work and time pressure, excessive workload, role conflict, role ambiguity, emotional labor, and poor physical working conditions that force the capacity of the employees and create stress on them can be given as patterns of job demands (Hakanen and Roodt, 2010). On the other hand, job resources represent “those physical, psychological, social, or organizational aspects of the job that may (a) reduce job demands and the associated physiological and psychological costs, (b) are functional in achieving work goals, and (c) stimulate personal growth, learning, and development” (Demerouti et al., 2001, p. 501). Social support, participative decision-making, psychological empowerment, autonomy, economic or career-related promotion availability, and job security are job resources (Hakanen and Roodt, 2010; Tummers and Bakker, 2021). The model suggests that job demands, and resources are negatively related (Bakker and Demerouti, 2007). In other words, while job demands are associated with burnout and adverse health outcomes, job resources are associated with WE and positive effects such as performance and commitment (Demerouti et al., 2001; Schaufeli and Bakker, 2004; Bakker and Demerouti, 2007; Hakanen and Roodt, 2010; Yumuşak et al., 2013), and JS (Bakker and Sanz-Vergel, 2013; Nguyen et al., 2018).

Past research conducted in the healthcare industry explained that the link between WE and JS grounded in the JD-R model (Nguyen et al., 2018; Gong et al., 2020). However, although the positive relationship between WE and JS has been mostly confirmed by previous studies (De Simone et al., 2018; Côté et al., 2021; Ng et al., 2021), it has been found that there are studies that differ from the general pattern in recent studies. More specifically, we noticed that the studies conducted during the COVID-19 period are inconsistent with previous studies (Gimenez-Espert et al., 2020; Stover, 2020). To clarify this inconsistency, the following research questions were tried to be answered with the meta-analysis method:

*RQ*_1_*:* What is the amount of overall effect size?
*RQ*_2:_ What is the direction of the overall effect?
*RQ*_3_*:* Is the overall effect homogeneous or heterogeneous?
*RQ*_4_*:* What is the statistical power of the overall effect size?

Although job and personal resources are the basis for WE and, in turn, positive individual and organizational outcomes, the job resources-work engagement-positive outcomes sequence may be moderated by the job demands (Bakker and Demerouti, 2007). During the ongoing COVID-19 processes, job demands for healthcare professionals have gone beyond the ordinary, causing many healthcare professionals to be exhausted and quit their jobs (Yáñez et al., 2020; Ardebili et al., 2021; Zhang et al., 2022). Factors such as working under the risk of disease, long and exhausting working hours, excessive workload, inability to control the virus, hopelessness, and being forced to work have caused significant damage to the well-being of healthcare workers (Franza et al., 2020; Hacimusalar et al., 2020; Yildiz and Elibol, 2021; Babapour et al., 2022). Based on the explanations above, it has been predicted that the difficult working conditions in the COVID-19 process reduce healthcare professionals' engagement and JS. In this context, the following research question was formulated as follows:

*RQ*_5_*:* How does the presence of COVID-19 affect the relationship between WE and JS.

Past research found that engagement-related studies differ from Hofstede's (1983) national culture dimensions. A recent meta-analysis (Yildiz et al., 2021) conducted on nurses demonstrates that individualism and long-term orientation as subdimensions of national culture moderated the study results. More specifically, researchers found that the level of WE in individualistic cultures is higher than in collectivistic cultures (Hu et al., 2014; Mazzetti et al., 2021). Mazzetti et al. (2021) explained these results as employees in western culture have more opportunities to identify themselves with their jobs and develop their competencies. On the other hand, in collectivist cultures, it is explained as more self-sacrifice of employees to accomplish common goals instead of their personal goals or needs. Considering the arguments, the following research question was determined:

*RQ*_6_*:* How does the national culture dimensions affect the relationship between WE and JS.

## Methods

“Meta-analysis is the statistical combination of results from two or more separate studies. Potential advantages of meta-analyses include an improvement in precision, the ability to answer questions not posed by individual studies, and the opportunity to settle controversies arising from conflicting claims” (Deeks et al., 2019: p.241). This meta-analysis was conducted according to Hunter and Schmidt (1990) approach. Firstly, the overall effect size was calculated. Secondly, heterogeneity (i.e., τ^2^, *I*^2^) among the studies was calculated (Cochran, 1954; Higgins and Thompson, 2002; Viechtbauer, 2005; Riley et al., 2011). Finally, moderator analyses were conducted to determine which factors caused the heterogeneity.

Before conducting analyses, a series of influence tests were performed to detect whether there are outliers influencing study results abnormally or not (Viechtbauer and Cheung, 2010; Sterne et al., 2011; Sedgwick, 2013). Rank correlation analyze (Begg and Mazumdar, 1994) and Egger's regression analyze (Egger et al., 1997; Sterne and Egger, 2006) were performed to check for funnel plot asymmetry. Also, the fail-safe *N* test was conducted for the presence of a file drawer problem (Rosenthal, 1979).

The analysis was performed using R (version 4.1.1) (R Core Team, 2020) and the “psych” (version 2.2.5) (Revelle, 2022), “metapower” (version 0.2.2) (Griffin, 2020), “metafor” (version 3.4.0) (Viechtbauer, 2010), “robumeta” (version 2.0) (Fisher et al., 2017), “dmetar” (version 0.0.9) (Harrer et al., 2019), “psychmeta” (version 2.6.3) (Dahlke and Wiernik, 2019), and “meta” (version 5.2.0) (Balduzzi et al., 2019) packages.

### Design and search methods

To formulate research questions, Population, Intervention, Comparison, and Outcome (PICO) framework, which is widely used in systematic literature reviews in the health field, was used (Kang et al., 2020; Salari et al., 2020; Lulli et al., 2021; Schiavenato and Chu, 2021). In this study, the PICO framework was identified as follows:

Population: Healthcare workers,
Intervention: Presence of COVID-19,
Comparison: The level and direction of the relationship between WE and JS (Before COVID-19 and during COVID-19),
Outcomes: Quality of the relationship between study variables (overall effect size and conditional effect of COVID-19 on the overall effect size).

**Figure 1:**
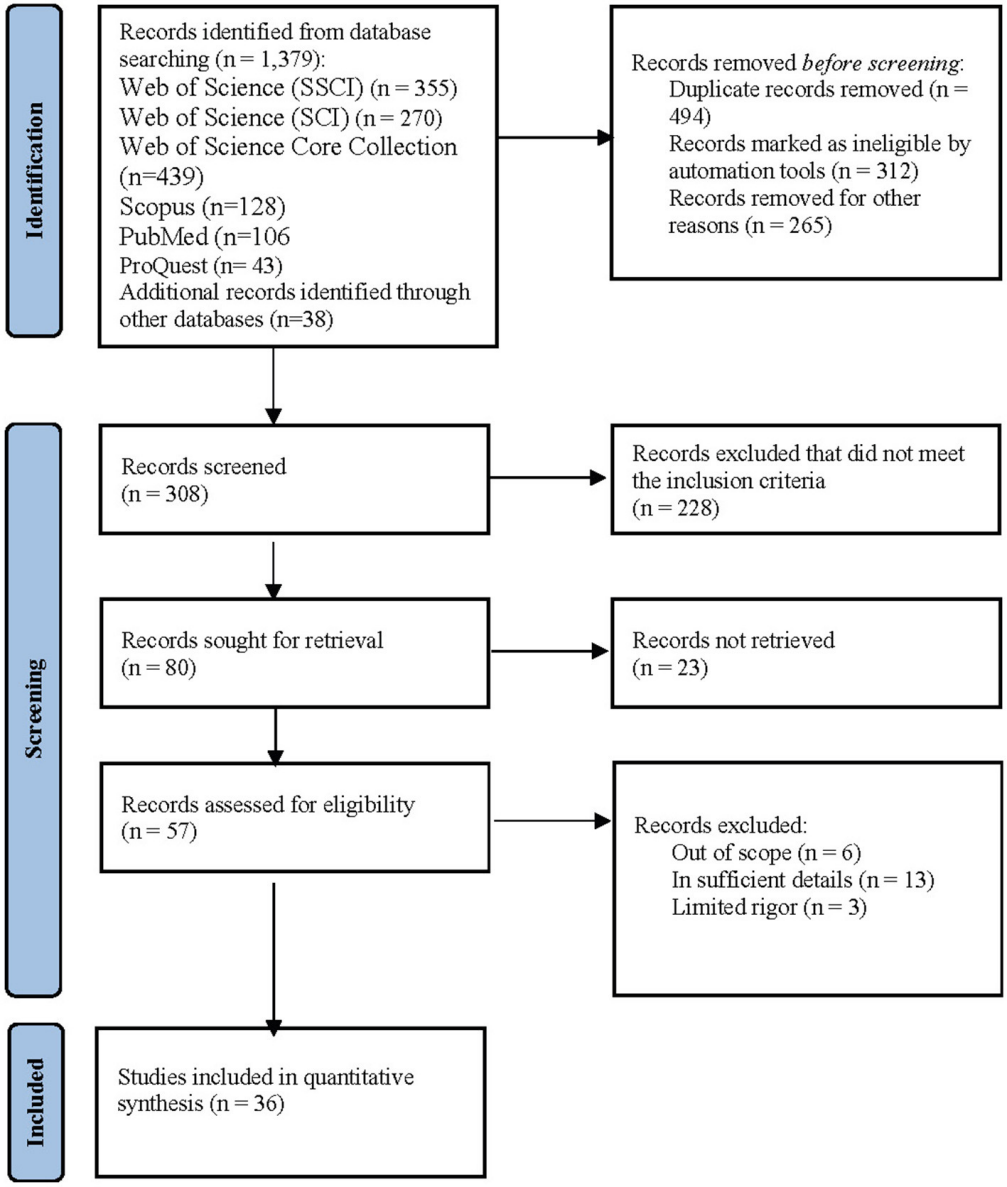
PRISMA flow diagram.

Preferred Reporting Items for Systematic Reviews and Meta-Analyses (PRISMA) followed the reporting of the process and results of this meta-analysis (Mother et al., 2010; Page et al., 2021). As illustrated in Figure 1, Web of Science (WoS), Scopus, PubMed, ProQuest, and other resources (e.g., Google Scholar and other databases in English) were searched up to June 30th, 2022. EndNote (v. 20) reference management tool was the main search instrument for the key search terms (“PHYSICIAN” OR “NURSING” OR “NURSE” OR “DOCTOR” OR “MEDICAL OFFICER” OR “MIDWIFE” OR “HEALTH OFFICER” OR “MEDICAL PRACTITIONER” OR “GENERAL PRACTITIONER” OR “MEDICAL DOCTOR” OR “HEALTH” OR “HEALTHCARE”) AND (“WORK ENGAGEMENT” OR “ENGAGEMENT”) AND (“JOB SATISFACTION” OR “SATISFACTION”) (see Table 1 for search results). All databases were queried with the same set of keywords. As a result of the identification process, 1,379 publications were reviewed in Jan 2022. The collected secondary data were examined by using the R statistical language (R Core Team, 2020), which requires advanced coding knowledge and has personalized graphics and display options (Yildiz et al., 2021; Yildiz and Yildiz, 2022).

**Table 1:** Summary of studies.

	**Researchers and publication year**	**Sample size**	**Type of facility**	**Occupation/position**	**Country**	**Quality score**
1	Artha and Piartrini (2021)	159	Hospitals	Nurses	Indonesia	10
2	Côté et al. (2021)	289	Medical clinic	Healthcare workers	Belgium	14
3	Ge et al. (2021)	1.327	Health services	Healthcare workers	China	11
4	Richert (2021)	100	Hospital	Nurses	USA	10
5	Mukaihata et al. (2022)	446	Hospital	Nurses	Japan	13
6	Ng et al. (2021)	279	Hospital	Healthcare workers	Malaysia	12
7	Sarfaraz et al. (2022)	305	Medical university	Healthcare workers	Pakistan	12
8	Gimenez-Espert et al. (2020)	92	Hospital	Nurses	Spain	11
9	Gong et al. (2020)	370	Hospital	Nurses	China	10
10	Jinmei et al. (2020)	418	Hospital	Nurses	China	12
11	Lida et al. (2021)	175	Hospital	Nurse	Japan	9
12	Lin et al. (2020)	1.404	Health services	Healthcare workers	China	11
13	Orgambídez et al. (2020)	321	Hospital	Nurses	Spain	12
14	Stover (2020)	77	Hospital	Nurses	USA	11
15	Zhang et al. (2020)	2.693	Rural clinics	Village doctors	China	12
16	Ding et al. (2019)	282	Hospital	Nurses	China	11
17	Edwards-Dandridge (2019)	155	Hospital	Nurses	USA	11
18	Yan et al. (2019)	316	Hospital	Nurses	China	10
19	Khadhuri (2018)	677	Hospital	Nurses	UAE	13
20	Al-Sheyab (2018)	150	Hospital	Nurses	USA	12
21	De Simone et al. (2018)	194	Hospital	Nurses	Italy	8
22	Yan et al. (2018)	356	Hospital	Nurses	China	9
23	Zhang et al. (2018)	2.426	Hospital	Healthcare inspectors	China	10
24	Cooke (2017)	160	Hospital	Nurses	USA	10
25	Nguyen et al. (2018)	220	Hospital	Nurses	Australia	10
26	Noblet et al. (2017)	516	Australian community health services	Healthcare workers	Australia	12
27	Orgambídez-Ramos and de Almeida (2017)	215	Hospital	Nurses	Portugal	11
28	Pohl and Galletta (2017)	323	Hospital	Nurses	Belgium	10
29	Varghese (2017)	299	Hospital	Nurses	USA	12
30	Baumgardner (2014)	275	Hospital	Nurses	USA	12
31	Mache et al. (2014)	123	Surgery medicine	Surgery clinicians	Germany	12
32	Hussein (2013)	100	Hospital	Nurses	Egypt	10
33	Walker and Campbell (2013)	96	Hospital	Nurses	Australia	9
34	Jenaro et al. (2011)	412	Hospital	Nurses	Spain	10
35	Giallonardo et al. (2010)	170	Acute care setting	Nurses	Canada	10
36	Simpson (2007)	167	Hospital	Nurses	USA	12

### Inclusion and exclusion criteria

The literature review was conducted in line with a search strategy. The authors identified the inclusion and exclusion criteria according to the research questions designed according to the PICO framework. In this context, research met the following criteria included in the study:

(1) The publication must be written in English.
(2) The publication must have reported the correlation coefficient WE and JS.
(3) If there is no correlation coefficient, it must have reported the values (e.g., beta coefficient) to calculate the correlation coefficient.
(4) The publication must have been done on healthcare workers.
(5) The concepts must have measured with measurement tools in accordance with the definitions in the literature.

On the other hand, research that did not meet the following criteria was excluded from the study:

(1) The publication is written in other languages.
(2) The publication has not had sufficient statistics (e.g., correlation coefficient, beta values).
(3) Publications whose authors were reached by e-mail, but the necessary data were not provided.
(4) Publications in which the sample does not only consist of healthcare professionals,
(5) Studies that have not been handled with a quantitative method such as a book, book chapter, editorial, content analysis, or qualitative studies.

### Coding process and quality appraisal

Two authors reviewed a total of 1,379 publications. As previously mentioned, we followed the PRISMA flow diagram (Page et al., 2021) that consists of three stages, namely identification, screening, and included (see Figure 1). After a detailed review comprised of three stages, the data of 36 individual studies eligible for the analysis were coded into an Excel file. Firstly, Cohen's (1960) weighted Kappa correlation test was performed to test inter-rater reliability. Accordingly, the two authors coded a randomly selected study independently, and then the codes were assessed as two vectors in the R environment. Accordingly, Cohens' Kappa correlation coefficient was calculated as *r* = 0.85, which is robust. Further, to evaluate the quality of the coded studies, we used Cicolini et al.'s (2014) tool, which is “Quality Assessment and Validity Tool for Correlational Studies”. The tool has thirteen criteria for evaluating the design, sample, measurement, and statistical analysis of the included studies. The total score of the tool is 14 and classified into three sub-categories, namely low (0–4), medium (5–9), and high (10–14). As a result of the quality assessment process, the quality scores of the publications were determined as high (*k* = 32), medium (*k* = 4), and low (*k* = 0), respectively (see Table 1). Finally, the presence of COVID-19 variable was determined as a categorical variable, depending on whether the data of the studies in the publication pool were collected during the COVID-19 pandemic. On the other hand, the international cultural dimensions of the countries where the data of the studies in the analysis were collected were based on Hofstede's classification (Hofstede, 2022). According to this classification, the culture scores of the countries were in the range of 0–100 and were coded into the data set as secondary data.

### Study characteristics

**Figure 2:**
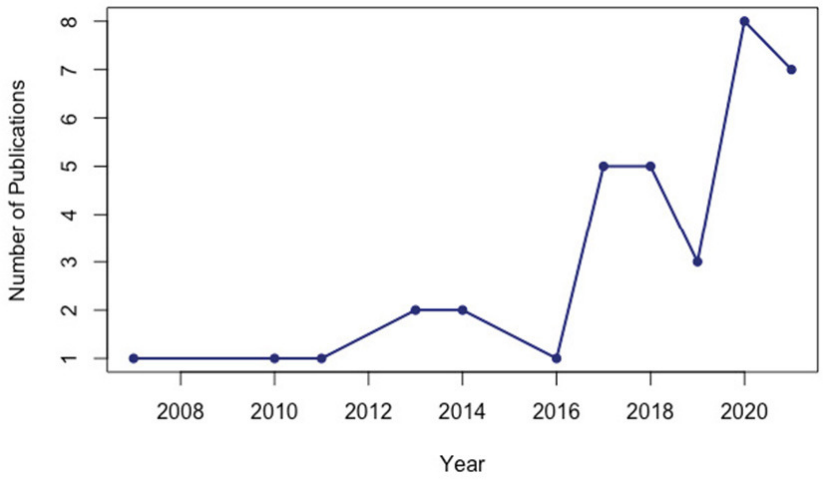
Annual scientific production.

**Figure 3:**
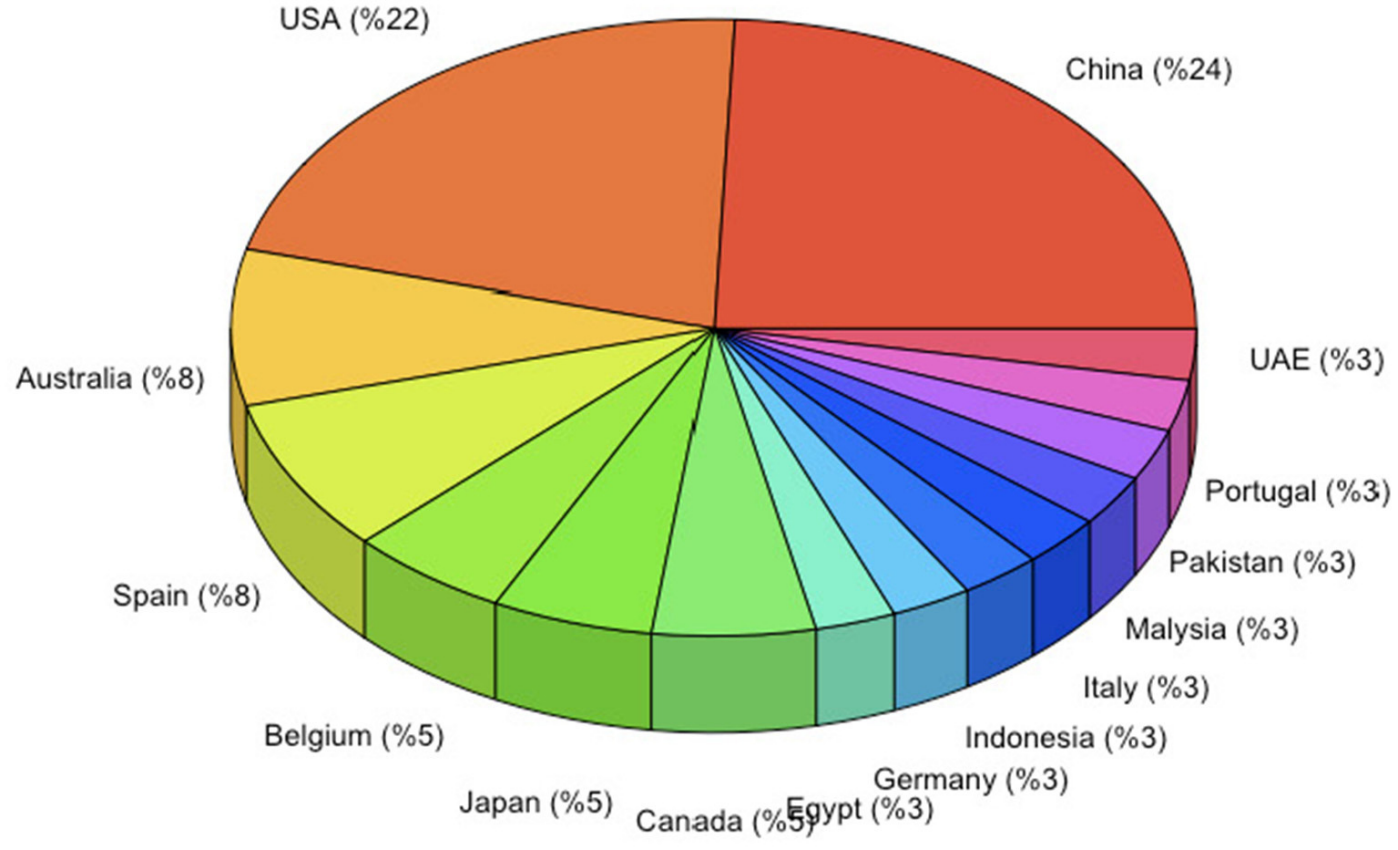
Distributions of the publications by countries.

The total sample size of 36 studies consisted of 16,087 (Mean= 447 ± 595) healthcare professionals who primarily work in hospitals. As illustrated in Figure 2, studies that meet the inclusion criteria were published between 2007 and 2021. While with the 8 publications, 2020 is the most productive year, it is noteworthy that the number of studies examining the relationships between WE and JS dramatically increased after 2016. As seen in Figure 3, most of the reviewed publications were carried out in China (%24) and the USA (%22), followed by Australia (%8) and Spain (%8). As World Health Organization (2022) suggests there has not been any country where healthcare workers were not seriously and adversely influenced by the COVID-19 pandemic. The meta-analysis draws on studies from a vast geography. The detailed information about the reviewed studies is presented in Table 1.

## Results

### Data and publication bias

**Figure 4:**
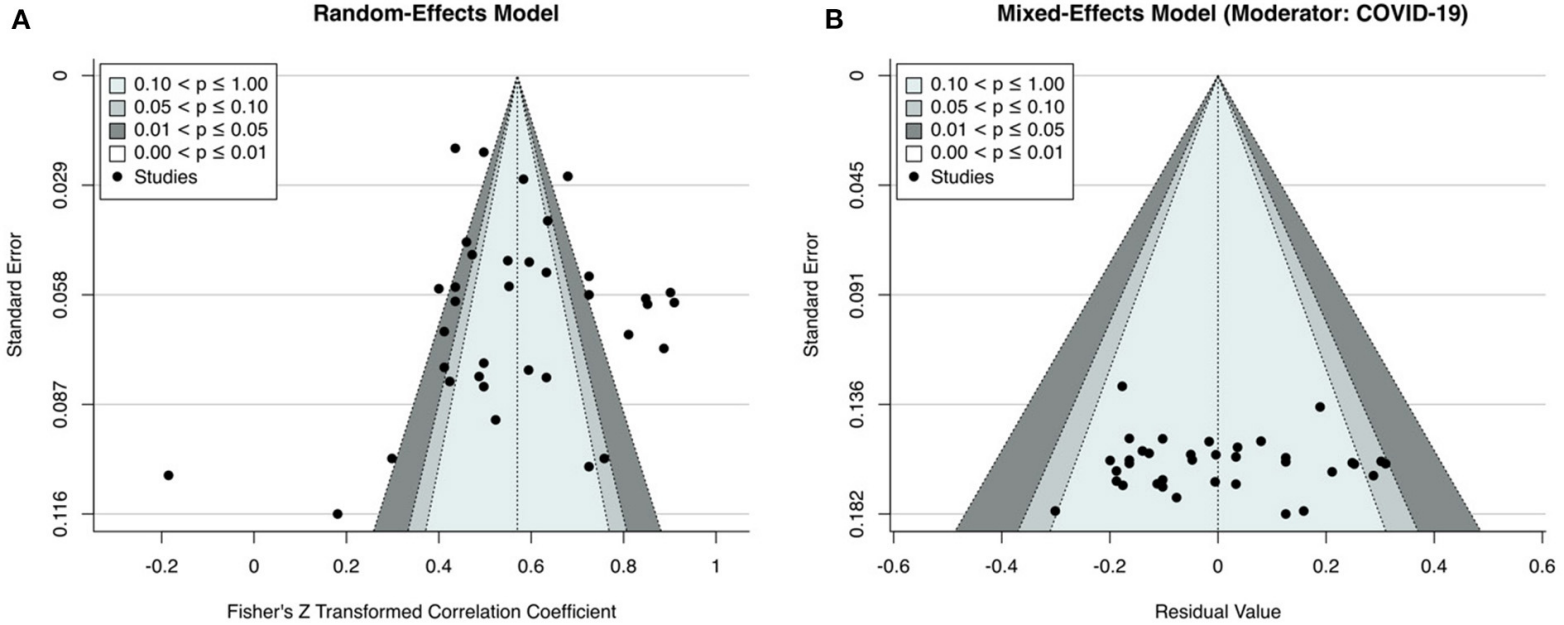
**(A,B)** Funnel plots.

Findings showed that 36 studies' amount of heterogeneity is above *I*^2^ = 75% categorized as a high level of heterogeneity [Q _(35)_ = 358.78, *p* < 0.01, τ^2^=0.04, *I*^2^ = 94.07%] (Higgins and Thompson, 2002; Borenstein et al., 2010). The *I*^2^ statistic indicates that the source of heterogeneity is stemming from true heterogeneity, not from the sampling error (Borenstein et al., 2010). Because the level of heterogeneity is high random effect model was used in the meta-analysis (Hedges and Vavea, 1998). Further, influence tests, Cook distance, and Baujat plot indicated that there were no overly influential studies in the publication pool. The result of the Fail-safe N test showed that 53.613 extra publications which have negative or inconsistent results might change the current research results. Figures 4A,B illustrates the funnel plot distribution of random-effect model and mixed effect model respectively. Rank correlation and Egger's regression tests also revealed that the studies in the funnel plot did not show an asymmetrical distribution (*p* = 0.69 and *p* = 0.12, respectively). Based on these explanations results were considered as robust.

### Meta-analytic results

**Figure 5:**
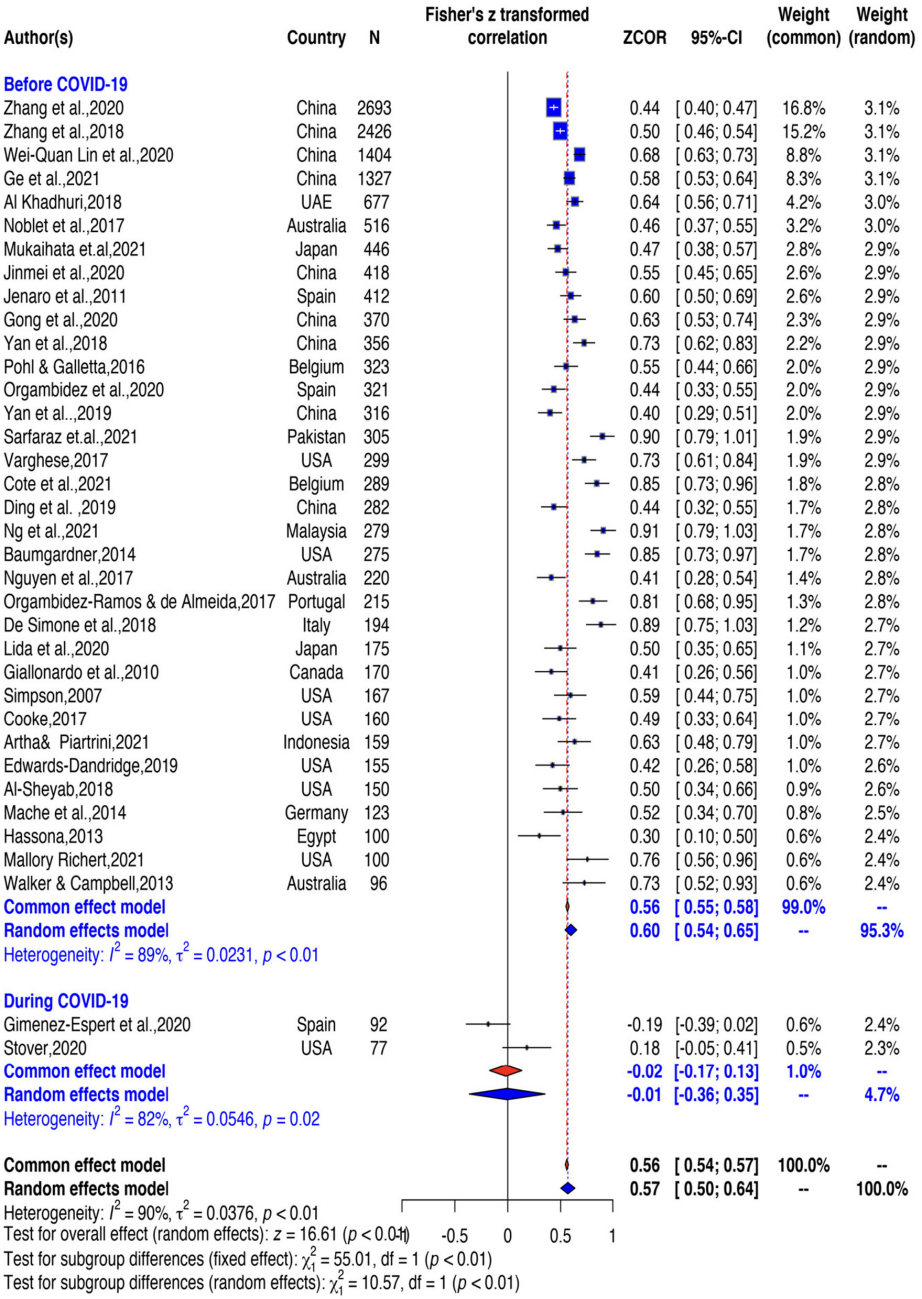
Forest plot.

A total of 36 studies were synthesized within the scope of the research. Although most of them are positive (97%), Fisher's r-to-z transformed correlation coefficients of individual studies ranged from −0.1851 to 0.9097. As a result of the meta-analysis run by choosing the random effect model, the average effect size was found to be μ =0.57 (95% CI: 0.50–0.64), which is positive and differed significantly from zero (*z* = 16.612, *p* < 0.0001) (see Table 2). Based on the sample size and correlation values of each study, the weighted effect sizes and the overall effect size created by these individual effect sizes are presented in the Forest Plot as both the general and the subgroups of the COVID-19 (before COVID-19 and during COVID-19 variable (see Figure 5).

**Table 2:** Meta-analytic results.

**Relationship**	***k***	***N***	** $\bar{r}$ **	***z***	**95% CI**	**80% CV**	***Q***	***I*^2^ *(%)***	**Egger's *t-*test**	**Failsafe-*N***	**Power**
Work Engagement and Job Satisfaction	36	16.087	0.57*	16.612	[0.50, 0.64]	[0.36, 0.46]	357.776	94.07	−1.57	53.613	1

K, number of independent samples, N, total sample size, r, correlation effect size, 95% CI, lower and upper bounds of the 95% confidence interval for $\bar{r}$; 80% CV, lower and upper bounds of the 80% credibility value for $\bar{r}$; Q, weighted square deviation; I^2^, proportion of true variance relative to total variance; ^*^p < 0.001.

### Moderator analyses

**Figure 6:**
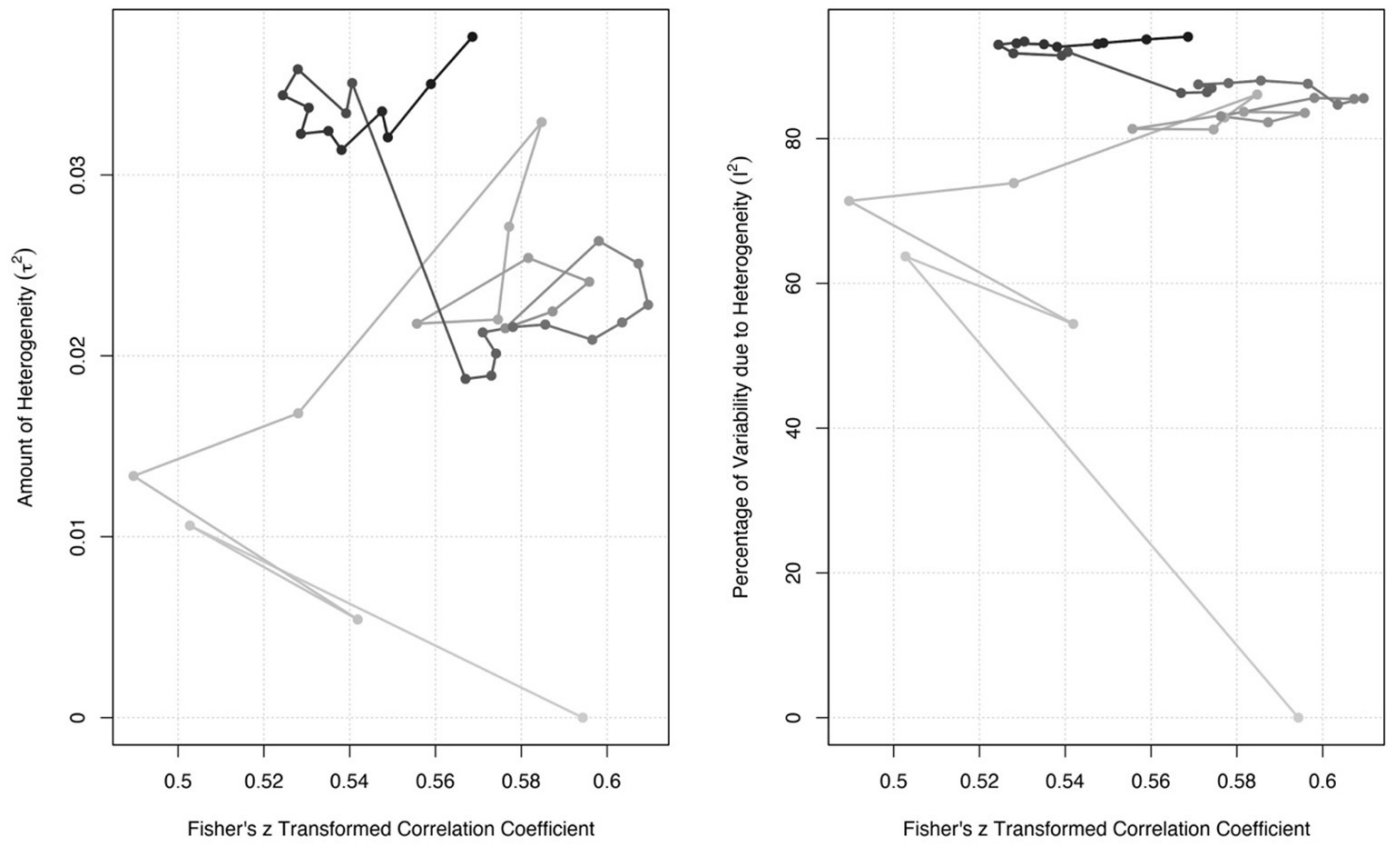
The plot of cumulative results by years.

As can be seen in Figure 6, although the average effect size of the link between WE and JS has decreased compared to previous years, the heterogeneity levels of the studies have increased dramatically. To explain the cumulative temporal variability, the light color of the figure or dots in the figure indicates the beginning years, while the dark colors indicate the last years.

**Figure 7:**
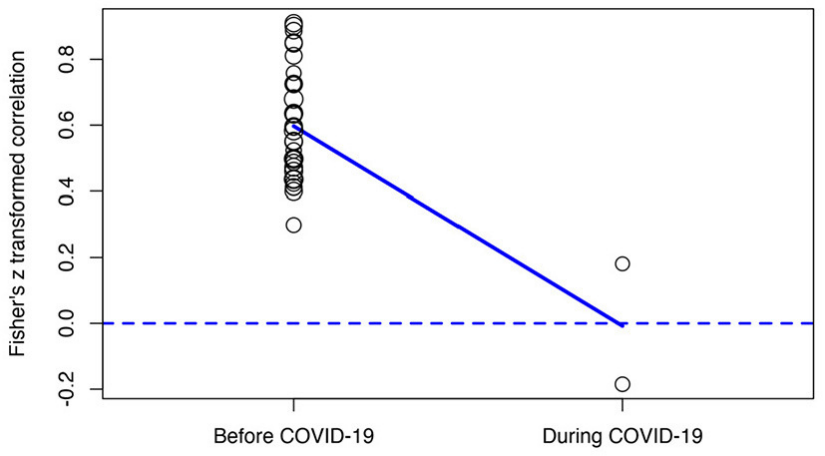
Meta-regression performed with COVID-19 process as moderator.

There was a high heterogeneity in the included effect sizes [Q _(35)_ = 358.78, *p* < 0.01, τ^2^=0.04, *I*^2^ =94.07%] for the correlation between study variables. To explain overall effect size heterogeneity moderator and subgroup analyses were run. Moderator analysis showed that the moderating effect of national culture was found as non-significant [Q_M__(6)_ = 11.1430, *p* = 0.0841]. On the other hand, it was found that presence of covid 19 significantly moderated the link between WE and JS [Q_M__(1)_ = 19.4289, *p* < 0.0001]. Because the total number of studies is 36 which is above K > 9 we conducted subgroup analysis (Harrer et al., 2021). As seen in Table 3 and Figure 7, studies are categorized into two subgroups under the data presence of COVID-19 variable namely No (before COVID-19) (*k* = 34) and Yes (during COVID-19) (*k* = 2). When the overall effect size (*r* = 0.57, 95% CI [0.50, 0.64], *p* < 0.001) was compared with the subgroups effect sizes namely No (before COVID-19) (*r* = 0.60, 95% CI [0.54, 0.65], *p* < 0.001) and Yes (during COVID-19) (*r* = - 0.01, 95% CI [−0.36, 0.35], p>0.05), the difference between the subgroups effect size was statistically significant (*p* < 0.001). Such that, while the correlation is small and negative (*p* > 0.05) during COVID-19, it is higher and positive before the COVID-19. Presence of COVID-19 explained 37.08% of effect size heterogeneity.

**Table 3:** Moderating effect of COVID-19 and subgroup analyses.

**Moderator**	***k***	***r***	**ß**	**95%CI**	***P*_subgroup_**
Presence of COVID-19			−0.60	[−0.87 to −0.34]	0.0011
No	34	0.60		[0.54 to 0.65]	
Yes	2	−0.01		[−0.36 to 0.35]	

k, number of included effect size estimates; r, Pearson correlation coefficient; ß, meta regression coefficient from a model in which a categorical moderator with two levels was entered as a predictor; 95% CI corresponds to the ß coefficient for moderators or the r values for individual moderator levels; p corresponds to the ß coefficient for moderators or the subgroup analysis for individual moderator levels.

### Power analysis

**Figure 8:**
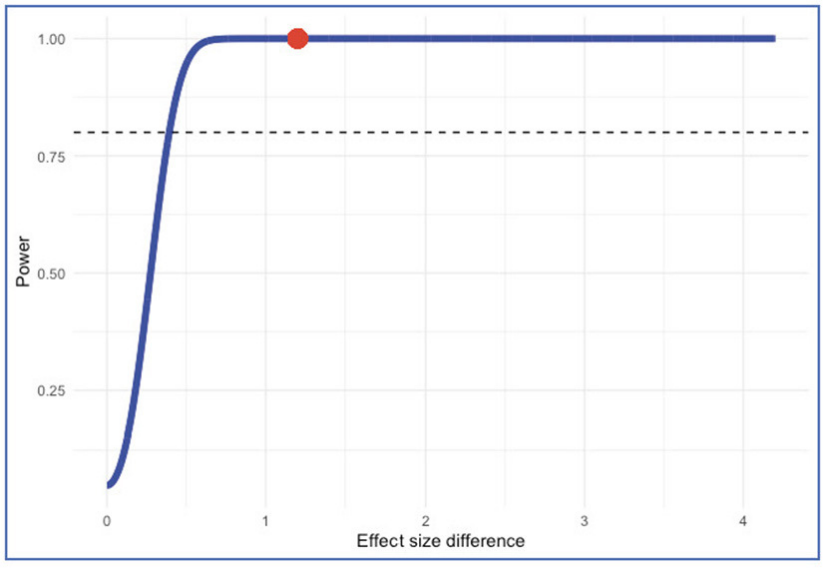
The statistical power of the subgroup analysis.

Because most of the meta-analyses were conducted on a small number of individual studies calculating and reporting the power statistic of the meta-analysis is important in terms of minimizing Type 1 error (Borenstein et al., 2021; Cuijpers et al., 2021; Griffin, 2021; Harrer et al., 2021). Despite the number of included studies being k= 36 in this study, to check the robustness of the statistical power we performed power analysis by using the “metapower” package (Griffin, 2020). Accordingly, given the pooled effect size of 36 individual correlation is *r* = 0.57, the average sample size is 447, and a large level of heterogeneity (*I*^2^ = 94.07%), the estimated statistical power was calculated as 1 out of 1 is robust. Additionally, the power of moderator analysis was calculated based on the effect sizes of two groups namely before COVID-19 (*k* = 34, *r* = 0.60) and during COVID-19 (*k* = 2; *r* = −0.01). Power of the moderator analysis is 0.99 out of 1. Lastly, power of the subgroup analysis showed that the minimum effect size difference required for sufficient power is 0.393. Accordingly, power for subgroup difference test (two-tailed) indicated that the power of subgroup analysis is 100% (Figure 8). Taken together, the results indicated that the statistical power of the meta-analysis is robust.

## Discussion

The relationship between WE and JS has been the focus of many studies. There has been consistent academic and policy interest in this relationship. Theoretically, the relationship between WE and JS is often considered settled to be positive. Yet, most studies that find such a positive relationship examine relatively stable and supportive work environments. There is a need for exploration of this relationship in different turbulent, dynamic, and adversarial contexts. The COVID-19 pandemic provides such a toxic context specifically for healthcare workers. In the volatile context of the COVID-19 pandemic, we question whether the positive relationship between WE and JS still holds a positive relationship. Our findings based on a meta-analytical study reveals counterintuitive findings that the relationship between WE and JS turns negative for healthcare workers in the context of the COVID-19 pandemic. We interpret this finding to suggest that the specific macro context shapes workplaces' beliefs, feelings, and behaviors. In a dramatic context, such as COVID-19 pandemic, where WE is not encouraged but enforced for healthcare workers, JS is adversely affected. Ardebili et al. (2021) stated that in the ongoing COVID-19 pandemic, excessive work demands for healthcare professionals brought along the lack of job resources, causing employees to lose control over their work.

During the pandemic, healthcare workers who worked on the front line, regardless of their temporal engagement, were the ones most at risk (Zhang et al., 2021). In this process, the balance between their work and life was disrupted, and their psychological health and well-being were adversely affected (Giorgi et al., 2020). This view is also consistent with the Job Demands-Resources (JD-R) model (Demerouti et al., 2001; Bakker et al., 2008; Schaufeli and Taris, 2014; Schaufeli, 2017). According to the JD-R model, insufficient job resources (supervisor support, autonomy, coaching, harmony, coaching, team cohesion, and colleague support) cause burnout, a decrease in positive psychological resources, and consequently a decrease in performance (Bakker et al., 2008). Therefore, it is not surprising in terms of theoretical expectations that a challenging process such as the COVID-19 pandemic turns the relationship between two positive variables, such as WE and JS, into neutral or negative. While this theoretical finding accounts for the relationship between the research variables, the study also has significant policy implications.

Most policy interventions focus on improving and strengthening WE. However, as in our study, policy interventions in a context where WE is regulated with coercive measures, JS declines. As highlighted in previous studies, insufficient job resources “precludes actual goal accomplishment, which causes failure and frustration” (Bakker et al., 2003; Bakker et al., 2008: p. 3). Similarly, past research also addressed that to protect their positive resources; employees are more likely to exhibit withdrawal behaviors and lower levels of commitment when the level of job resources supported by the organizations is low (Demerouti et al., 2001; Bakker et al., 2003; Yildiz et al., 2017). Thus, policy interventions should seek to alleviate the negative consequences of the COVID-19 pandemic through institutional innovation (Palalar Alkan et al., 2022) and organizational support (Cicek et al., 2021; Greenhalgh et al., 2022). Such innovation could come in the form of improving conditions for WE even when draconian measures are taken at the national level to curb worker agency and power to take leave, breaks, and even preventative measures to protect themselves, their families, and the public (Greenhalgh et al., 2022).

Moreover, providing organizational trust to employees at all stages of the work together with organizational support can increase the positive psychological capital of employees and alleviate the negative effects on JS (Yildiz, 2019). In emergencies such as pandemics, employees to maintain the balance between their families and their jobs. During the pandemic, this balance has been disturbed by the possibility of the contagious disease being transmitted to other family members. It is very important to support employees who are struggling with excessive workload at a time of this risk (Pappa et al., 2020; Lulli et al., 2021). Future interventions should aim to reduce the risk of infection of frontline personnel and increase their self-confidence (Zhang et al., 2021). The conditional role of stress experienced by healthcare professionals during the pandemic should also be considered (Gómez-Salgado et al., 2021). The psychological distress felt while working in pandemic and excessive workload conditions may adversely affect the mental health of the employee and as a result may cause low WE and JS (Chen et al., 2020; Pappa et al., 2020; Zhang et al., 2021).

Protective measures should be offered to meet personal demands and needs, such as flexible hours, dynamic workload management, employee allowances, motivational speeches from dedication leaders, effective leadership, a strong collaborative atmosphere, fast and easy transport to home, and alternative accommodation for employees who do not want to go home to prevent contagion after the shift (Chen et al., 2020; Kisely et al., 2020; Pappa et al., 2020; Walton et al., 2020; Gómez-Salgado et al., 2021). These measures create a more humane working environment, increase the psychological well-being of employees, and increase JS by enabling employees to engage in work even under difficult conditions. Taking these measures can improve self-dedication and over time, the employee's intention to leave the job can be eroded. Conversely, without dedication, an employee is less likely to stay on the job for a long time. On the other hand, although previous studies have reported that national culture dimensions moderate the relationship between the research variables (Hu et al., 2014; Mazzetti et al., 2021), no significant moderating effect of national culture was found in this study. On the other hand, although previous studies have reported that national culture dimensions moderate the link between the study variables (Hu et al., 2014; Mazzetti et al., 2021), no significant moderating effect of national culture was found in this study.

## Conclusion

There are systematic reviews on JS and WE i.e., Harter et al. (2002), Halbesleben (2010), and Lu et al. (2019), this study shows that the COVID-19 pandemic has reversed the relationship between WE and JS. We explain that this could be due to several factors such as the intensification of work, and coercive work practices, specifically for healthcare workers during the COVID-19 pandemic (Yildiz et al., 2015, 2017). The findings could also signal a decline in satisfaction when WE is enforced by law and policy during the pandemic. The policy implication of our findings is that traditional people management interventions to strengthen WE to foster satisfaction may not work in the context of a pandemic. The positive effect of the trust provided by the organization on this relationship should also be considered (Bulińska-Stangrecka and Bagieńska, 2021). Policymakers need to co-design interventions with workers to balance the engagement needs of the sector and combat the negative consequences of coercive engagement on JS through co-design and innovation.

### Limitations and future research

Along with its strengths, this study has some limitations that can be addressed by future researchers. The pandemic process has reversed the positive relationship between WE and JS. Although we used national culture and presence of COVID-19 to determine the heterogeneity of the research results, the management of the pandemic varied by country, and these samples were also collected from workplaces operating under these conditions (Lulli et al., 2021). Therefore, this may be the reason why this positive relationship has turned into negative or neutral during COVID-19. Most of the studies were conducted on nurses; further research could be conducted on more heterogeneous samples that could strengthen the generalization of the study results. Although some of the studies had samples from a single health institution, the majority were obtained from more than one institution. This supports the generalizability of the studies, but it would be beneficial to conduct more studies in more than one health institution in the future. The fact that the selected studies are predominantly cross-sectional raises concerns about the strength of the results and therefore makes it difficult to draw causal inferences. Lack of standardization of measurement tools in the included studies and the differences in the sample selection method are other factors that may affect the research results. In addition, one of the inclusion criteria in this study was that publications should be in English. This criterion means that we did not synthesize the results of articles in other languages, and this may result in language bias (Scott et al., 2022). However, considering that the data of the included studies are from the Asian, Middle Eastern, European, and American health systems, it can be said that this situation does not create a language bias. To increase the power and holistic structure of this study, all studies in English were tried to be reached by using databases. Further research could synthesize larger datasets by include the results of studies in different languages.

The scope of this study is limited to healthcare workers and their WE and JS relationship. Future research could explore the innovative policies that could mitigate the negative consequences of the pandemic on WE and JS. Researchers in different regulatory environments could also examine whether organizational support in healthcare setting moderates the link between WE and JS. Because of the analytic nature of this study, qualitative studies were not synthesized. To reach a deeper understanding of the topic, further research could examine the topic with systematic reviews or content analysis. Because searched databases were limited to the specific ones mentioned in the study's earlier sections, further research could also search other databases such as national thesis databases and online university libraries. This study investigated the moderator roles of presence of COVID-19 and national culture on the effect size heterogeneity. In explaining effect size heterogeneity, moderators play an essential role in the meta-analyses; therefore, to explore the source of heterogeneity, further research could test other moderators' effects such as age, gender, tenure, salary, and profession, and type of publication. Finally, despite the statistical power of this study being high, we investigated 36 studies conducted on healthcare workers due to the limited amount of research. It is recommended that future researchers conduct new research at periodic times, especially after the pandemic and when more research piles are formed.

## Data availability statement

The raw data supporting the conclusions of this article will be made available by the authors, without undue reservation.

## Author contributions

BY, TY, HY, and MO contributed to conception and design of the study. BY and TY organized the database and wrote the first draft of the manuscript. BY performed the statistical analysis. HY, MO, and BY wrote sections of the manuscript. All authors contributed to manuscript revision, read, and approved the submitted version.

## Conflict of interest

The authors declare that the research was conducted in the absence of any commercial or financial relationships that could be construed as a potential conflict of interest.

## Publisher's note

All claims expressed in this article are solely those of the authors and do not necessarily represent those of their affiliated organizations, or those of the publisher, the editors and the reviewers. Any product that may be evaluated in this article, or claim that may be made by its manufacturer, is not guaranteed or endorsed by the publisher.
